# Liquid Vibration Energy Harvesting Device Using Ferrofluids

**DOI:** 10.3390/mi14081588

**Published:** 2023-08-12

**Authors:** Nia Hannon, Christopher W. Harrison, Marcin J. Kraśny, Daniel Zabek

**Affiliations:** 1 School of Engineering, Cardiff University, Cardiff CF24 3AA, UK; 2 Translational Medical Device Lab, Lambe Institute for Translational Research, College of Medicine, Nursing and Health Sciences, University of Galway, H91 TK33 Galway, Ireland

**Keywords:** energy harvesting, ferrofluid, vibration, liquid generator, mechanical energy scavenging

## Abstract

Mechanical vibrations can be effectively converted into electrical energy using a liquid type of energy harvesting device comprised of a ferrofluid and a permanent magnet-inductor coil assembly. Compared to solid vibration energy harvesting devices, the liquid nature of the ferrofluid overcomes space conformity limitations which allow for the utilization of a wider range of previously inaccessible mechanical vibration energy sources for electricity generation and sensing. This report describes the design and the governing equations for the proposed liquid vibration energy harvesting device and demonstrates vibration energy harvesting at frequencies of up to 33 Hz while generating up to 1.1 mV. The proposed design can continuously convert mechanical into electrical energy for direct discharge or accumulation and storage of electrical energy.

## 1. Introduction

Energy harvesting refers to the process where the energy that would otherwise remain unused or wasted is collected or scavenged from the environment for local use in electric and electronic applications. Harvesting energy offers a unique opportunity to power electronic devices where the replacement of batteries is impractical or difficult, such as to power sensors installed in remote places or in the human body [1]. Hence, energy harvesting systems are often small-scale, with power levels in the range of nanowatts to hundreds of milliwatts. For this reason, the primary application is to power small sensors, actuators, or wireless communication devices [2]. These sensors and actuators have many uses, such as condition monitoring of machinery in precision agriculture, healthcare, and smart cities [3]. Examples of energy sources that can be utilized in harvesting systems include motion, temperature oscillations and gradients, light, electromagnetic radiation, and chemical energy. There are numerous potential mechanical motion or vibrational sources which could be tapped for generating electricity, such as, e.g., breathing and pulsating hearts in the human body, vibration in machinery, oscillations in civil structures as well as natural mechanical forces such as wind, tidal waters, and acoustic waves [4]. These vibrations and motions can be harvested into electrical energy using the piezoelectric effect [5], electromagnetic forces [6], or electrostatic and triboelectric forces [7]. Among these options, electromagnetic forces based on Faraday’s law of induction have gained significant popularity for their scalability and efficiency in converting mechanical energy. In traditional electromagnetic harvesters, the alteration in induction, or flux linkage, comes from vibrations causing a movement of a permanent magnet in relation to an inductive coil. This coil is then connected to an external electronic circuit responsible for energy conversion and management [8]. Electromagnetic harvesting systems can be described by a mass-spring-damper model with fixed geometries changing magnetic flux in a confined geometry. However, due to the fixed geometry of traditional permanent magnet energy harvesting devices, only very specific vibration sources are accessible. In permanent magnet systems, a magnetic liquid, i.e., ferrofluid, can also change the magnetic flux. Ferrofluids consist of magnetic nanoparticles (most commonly iron oxide—Fe_3_O_4_) suspended in a non-magnetic liquid carrier [9]. Although the nanoparticles themselves are not ferromagnetic in the absence of an external magnetic field, the suspended particles exhibit macroscopic net magnetization when exposed to an external magnetic field. Unlike solid materials, the liquid nature of the ferrofluid overcomes space conformity limitations and allows for various magnetic designs utilizing previously inaccessible geometries for mechanical vibrations. One early contribution of ferrofluid-induced flux changes used for energy generation investigated the feasibility of an electromagnetic micro-power generator utilizing the sloshing of a ferrofluid column in a seismically excited tank [10]. Experimental work in the literature consisted of vibrations of a cylindrical container filled with a light hydrocarbon-based ferrofluid on an electrodynamic shaker. The shaker was able to supply seismic excitations of varying magnitudes and frequencies to the container. Two axially magnetized permanent magnets applied a permanent magnetic field across the container. When mechanically excited, this sloshing motion of the ferrofluid changed the time-varying magnetic flux. Subsequently, an electric current was induced within the inductive coil wrapped around the container generating approximately 1 mW of power. Similar experiments were carried out by Purohit et al. [11], Oh et al. [12], and Kim [13], generating 9 mV open circuit voltage at 5 Hz vibration frequency. Oh’s work also concluded that using an iron yoke around the magnets increased the electromagnetic field (EMF) efficiency. Subsequently, Alazmi et al. developed an analytical model looking at the electro-magneto-hydrodynamics of an electromagnetic ferrofluid-based vibratory energy harvester [14]. This nonlinear model was developed using perturbation methods for the case of primary resonance excitation of the first mode and a two-to-one internal resonance between the first two modes. The model can be used to predict the effect of changing certain design parameters on the generated voltage. In 2017, Seol et al. proposed a ferrofluid-based vibration energy harvester with a hybrid triboelectric-electromagnetic operating mechanism [15]. The triboelectric nanogenerator generates electrical energy from mechanical energy using contact electrification and electrostatic induction [16]. This type of energy harvesting device has been the subject of recent development due to its simple design and low-cost materials. The paper by Seol et al. discusses a hybrid harvesting device that combines a triboelectric and an electromagnetic generator. It was identified that abrasion over time is an issue with triboelectric nanogenerators. So ferrofluids were suggested due to the reduced physical stress on liquid–solid contacts compared to solid–solid contacts. The triboelectric and electromagnetic components were combined into a single device in the experimental setup. It was also found that the device had a wide operating frequency band, which means it has the potential to behave reliably with a variable stimulation source generating 2.1 mV from electromagnetic induction and 240 mV from triboelectric friction at frequencies below 7 Hz. According to Khairul et al., most of the work performed on the subject of ferrofluid energy harvesters investigated the effect of fluid quantity, container shape and size, magnetic field, and vibrational frequency and amplitude on output voltage [17]. Recent works include a 2018 paper by Liu et al., which discusses a more comprehensive finite analysis model for fully coupled computational analysis of a ferrofluid-based energy harvester [18]. The model was validated by the experimental results when the model accounted for coupling. The experiment also investigated the effect of changing the placement and orientation of the magnets placed on either side of the tank. The highest voltage configuration was when the magnets were placed horizontally across the tank with the north poles aligned in the same direction, and the coil wound horizontally across the tank. Other research has suggested the use of ferrofluids not as the transduction element but as a lubricant in solid magnet energy harvesters [19]. Other types of liquid-type energy harvesting devices utilize thermal gradients [20], which serve as an indirect way of vibration energy harvesting using various types of thermo-mechanical principles such as pyroelectric [21] and piezoelectric [22] effects. Utilizing mechanical vibrations from thermal gradients has been recently explored by Wang et al., which looks at developing an analytical model for electromagnetic induction in pulsating ferrofluid pipe flows [23], which has been validated against experimental data collected by Monroe et al. [24]. The experimental setup involves using a peristaltic pump to pulse ferrofluid through a solenoid. The results show a maximum voltage output of 0.9 mV at 30 Hz. However, problems with this device arise when thought is given to possible applications. Although there are cases of peristaltic pumps existing in nature, in biological pumping systems such as the human esophagus, ureter, bile duct, and small intestine, it is not feasible to employ an electromagnetic energy harvesting system of this nature within the human body [25]. Several common themes can be identified throughout the literature, namely the use of ferrofluids to overcome the difficulties associated with using solid magnets in harvesting devices and the acknowledgment that ferrofluid energy harvesters have a wide operating frequency band and so offer tunability. However, the set-ups employing electrodynamical shakers to slosh a ferrofluid column introduce a new problem of spatial restrictions, as the column’s dimensions dictate the frequencies under which it best operates. Utilizing pumping action does not present the same problem; however, current literature demonstrates microvolt levels of induced voltages. Still, it remains unknown how a device that requires a peristaltic pump can be employed for vibration energy harvesting. It is also noted that underdeveloped concepts and opportunities for further research exist. For example, most studies neglect to investigate the effect of an intermittent and variable vibration source. In addition, most papers do not address the feasibility of implementing such devices or offer potential applications. Hence, we propose a ferrofluid-based electromagnetic energy harvesting device utilizing the capillary action of a ferrofluid motion inside a narrow channel. The primary objective of our work is to design a scalable harvester capable of easy adaptation to various shapes. This work also reports on the assembly and experimentally tested performance of the proposed ferrofluid-based electromagnetic energy harvesting device covering a wide range of vibration frequencies.

## 2. Materials and Methods

We propose a liquid vibration energy harvesting device utilizing capillary action in a ferrofluid which changes magnetic flux linkage in an external permanent magnet-inductor coil assembly converting mechanical vibrations into an electrical voltage. Liquids exhibit capillary action, or spontaneous wicking, where the fluid column flows up (or through) a capillary when the adhesive forces between a liquid and tube are greater than cohesive forces within the liquid. In horizontal tubes, changes in hydrostatic energy can be neglected, which reduces the capillary system to the liquid-gas interface area, or meniscus, minimizing the total free energy of the liquid confined by the solid walls of the tube [26]. In circular horizontal tubes with a constant radius r [m], the liquid confined by the capillary tube walls exhibits a meniscus for:

(1) $$r<\sqrt{\frac{\gamma }{\Delta \rho \cdot g}}$$

governed by the surface tension γ [N·m^−1^], the mass density difference between the liquid and the gas Δρ [kg·m^−3^], and constant gravity g [m·s^−2^]. The position of the meniscus in the tube can be precisely tuned using an externally applied pressure equal to the interfacial tension force to hold the liquid in place or can be moved along the tube if the applied pressure is greater than the interfacial tension force of the liquid [27]. In order to introduce a flow of a liquid through the horizontal cylindrical capillary tube, the applied pressure *P* [N·m^−2^] defined by the force *F* [N] acting on the surface of the meniscus and multiplied by the cross-sectional area *A_meniscus_* = $2\pi \cdot r^{2}$ [m^2^] of the liquid-air interface follows:

(2) $$P=\frac{F}{2\pi \cdot r^{2}}$$

or any r defined in Equation (1). The applied pressure *P* moves the ferrofluid liquid plug through the capillary, locally changing density ρ and permeability *μ* (*μ = μ_r_* + *μ*_0*)*_ from an air to a ferrofluid in the capillary or ferrofluid to air, respectively. Subsequently, the meniscus with a surface area *A_meniscus_* and the liquid plug travel the length—*L* [m] displaces a volume inside the capillary from a low permeability in air (*μ_r_* = 1) to a high permeability of a ferrofluid (*μ_r_* = 7) [28]. This change in permeability can change the magnetic flux *B* [T] of a permanent magnet as follows [29]:

(3) $$B=\mu_{r}\cdot \mu_{0}\cdot H$$

with the applied magnetic field strength—H [A·m^−1^] and the permeability of free space $\mu_{0}$ introducing a transient change in magnetic flux in the capillary. When neglecting friction losses between the fluid and the wall of the capillary, any change in pressure translates into ferrofluid movement followed by local changes in permeability, which has the potential to locally change the magnetic flux dB. Subsequently, following Faraday’s law a voltage—*V* [Volts] is induced from the local change in the magnetic flux dB in a solenoid which follows:

(4) $$V=N\cdot A_{solenoid}\frac{dB}{dt}$$

with a *N*—number of turns and surface area *A_solenoid_*. Continuous changes in pressure, or mechanical vibrations, generate transient changes in flux followed by the transient voltage response defined for a fixed solenoid cross-sectional area *A_solenoid_*. However, the available cross-sectional area of the coil is greater than the cross-sectional area for the ferrofluid meniscus due to the capillary walls, for which a relative permeability of air (*μ_r_* = 1) is assumed. Hence, the effective transient change in magnetic flux d*B* for the specific area *A_meniscus_* and the solenoid area *A_solenoid_* included in Equations (3) and (4) is defined as follows:

(5) $$B=\mu_{r}\cdot \mu_{0}\cdot H{\cdot A}_{meniscus\;}+\mu_{r}\cdot \mu_{0}\cdot H\frac{A_{solenoid}-A_{meniscus\;}}{A_{solenoid}}$$

accounting for the difference in flux change in the capillary wall and induced voltage signal from the solenoid proportional to the change in magnetic flux $\frac{dB}{dt}$.

**Figure 1:**
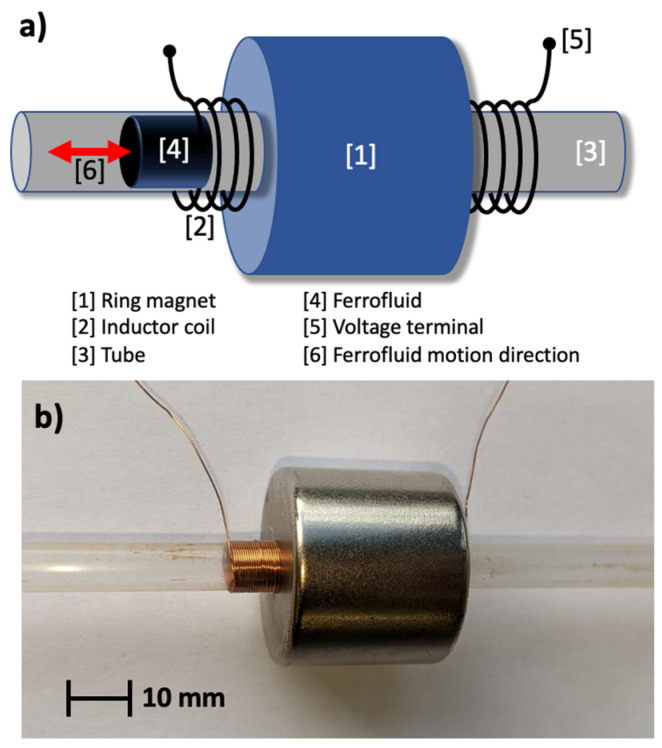
(**a**) illustration of a ferrofluid vibration motion energy harvester device with a ferrofluid plug in a coil/ring-magnet assembly and (**b**) picture of a ferrofluid energy harvester.

According to Equations (2) and (5), Figure 1a shows an illustration of a liquid vibration energy harvester device with a ferrofluid plug pushed into the coil/ring-magnet assembly and (b) a picture of the proposed ferrofluid energy harvester device. The energy harvesting device comprised a ferrofluid plug in a capillary tube, an external axially magnetized hollow cylindrical ring magnet, an inductor coil, and two inductor coil terminals connected to an external resistor. If an external pressure ∆P/∆t (Equation (2)) is applied to the capillary, local changes in density and permeability induced by the moving ferrofluid liquid plug create a relative change in the magnetic flux linkage dB/dt in the ring magnetic and coil (Equation (5)). Subsequently, the relative change in the magnetic flux linkage results in an induced voltage corresponding to Equation (4). Hence, every continuous oscillation of the fluid inside the capillary results in an AC voltage response which can be discharged across the external resistor as a current, converting mechanical vibrations into electrical signals. According to Ohm’s law, the product of generated voltage—*V* and electric current—*I* through the external resistor—*R* defines the electric power available from the externally applied pressure change. When the ferrofluid moves through the coil, a fraction of the kinetic energy is removed from the mechanical system and transferred into electrical energy. This novel mechanical approach to electrical energy conversion is based on the liquid nature of the ferrofluid, and the constant capillary force enables mechanical vibration energy harvesting by using external pressure changes.

Mechanical vibrations are available at various frequencies, and amplitudes form within the human body (breathing, blood pressure, walking), vehicles (aircraft, trains), structures, and industrial applications exhibiting a broad window of mechanical energy [4]. These vibrations can be converted into oscillating pressures through gases and liquids. In order to test and simulate vibrations in a controlled laboratory environment, a variable vibration source has been utilized to bench test the proposed liquid energy harvesting device for a frequency range between 1–33 Hz.

**Figure 2:**
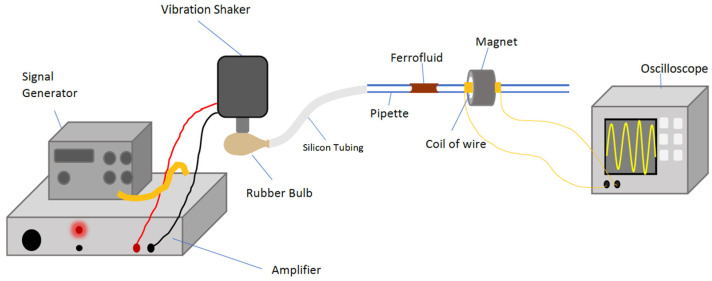
Experimental vibration test set-up for ferrofluid energy harvesting device comprises a pipette, ferrofluid, coil wire, and permanent magnet.

Figure 2 illustrates the experimental test set-up for a ferrofluid energy harvesting device comprised of a pipette, ferrofluid, coil wire, and permanent magnet. The variable frequency amplified vibrational shaker (LDS Permanent Magnet Vibration System V201) compresses a rubber bulb with a stamp that is attached to a pipette filled with a ferrofluid plug which is held stationary in the tube due to capillary action. The compression of the bulb introduces a pressure difference along the capillary pipette resulting in the ferrofluid plug being pushed along the pipette and through a coil of wire, acting as a pickup coil, and wrapped around a section of the pipette. As the bulb is reinflated, the ferrofluid plug is drawn back out of the pickup coil and returned to the equilibrium position. The energy harvesting device was mechanically decoupled from the shaker rubber bulb using a flexible silicone tube (length 1 m), so the energy harvesting device remained stationary while the fluid oscillated inside. The capillary pipette with an inner diameter of 3 mm and outer diameter of 4.5 mm was filled with 1 mL of Ferrotec EMG 707 at a concentration of 2% superparamagnetic Fe_3_O_4_ nanoparticles by volume suspended in water. The pipette was closed at the tip with glue so that the forward movement of the fluid acted as a piston compressing the remaining air in the pipette. The retraction of the vibration shaker armature released the compressed air spring, restoring the ferrofluid plug to the equilibrium position and reinstating the rubber bulb. The ferrofluid equilibrium position was set inside the coil so that excitation caused the plug to be pushed out of the coil. Even if the ferrofluid plug reaches different final displacements, the length that travels through the coil is constant due to the constant stroke of the shaker at different frequencies. Hence, only the speed of the plug changes with frequency and not the length. The coil around the pipette was wound with enameled copper wire (AWG 44) double-layer coil with an inner diameter of 5.5 mm and length *L* = 5 mm, resulting in ~200 turns and DC terminal resistance of 23 Ω. The coil was covered by an axially poled permanent ring-magnet (inner diameter 6 mm, outer diameter 23 mm) with a maximum magnetic field of 11 mT forming the coil/ring-magnet assembly of the energy harvesting device. The terminal voltage was measured with a National Instruments NI-PXI-4461 card in a NI-PXI-1033 (National Instruments, Austin, TX, USA) remote controller chassis at a sampling rate of 2 kHz (labelled ‘Signal Generator’ and ‘Oscilloscope’ in Figure 2) using LabVIEW ver. 2019 with a measurement acuity of ± 3% while employing a low pass filter with a cutoff frequency of 50 Hz.

## 3. Results and Discussion

The ferrofluid energy harvesting device was exposed to various sinusoidal pressure changes, and the transient open circuit voltage response was measured using the above experimental configuration. The results discussed are limited to a steady state operation of the energy harvesting device for a minimum of five minutes in order to eliminate potential start-up characteristics.

**Figure 3:**
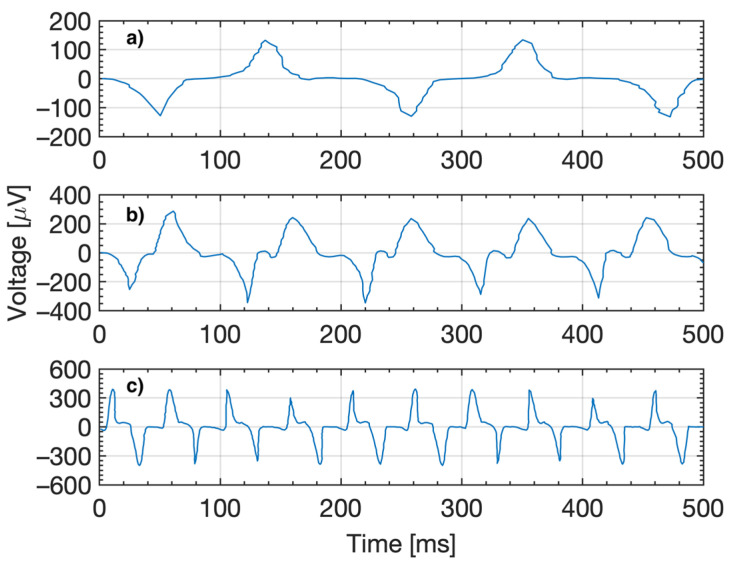
Transient voltage signal from the energy harvesting coil terminals at 5 Hz (**a**), 10 Hz (**b**), and 20 Hz (**c**) drive frequency.

Figure 3a shows the transient voltage signal from the coil terminals at 5 Hz vibration pressure drive frequency. The voltage has one maximum peak and one minimum with a peak-to-peak AC voltage—*V_PP_* of 280 µV for every cycle of the induced sine waveform; this represents the movement of the fluid out of the coil followed by the movement in the opposite direction back into the coil. According to Faraday’s law, the direction of the induced current is to oppose the change of flux that induced it. For this reason, the forward and back motion produced a positive and negative voltage amplitude. Figure 3b shows the transient average voltage signal at a 10 Hz drive frequency of 527 µV. At the turning point where the ferrofluid changes direction and moves back into the coil, the signal shows small voltage transient ripples. This can be attributed to the change in velocity and direction not being a smooth process, whereas, during the motion out of and into the coil, the ferrofluid travels smoothly, resulting in a smooth signal through the peaks but a transient signal around the voltage polarity switch. The general shape of the signal is replicated through the different frequencies between 1–30 Hz with very consistent peak-to-peak voltages for low frequencies of <4 Hz and higher frequencies of >10 Hz.

**Figure 4:**
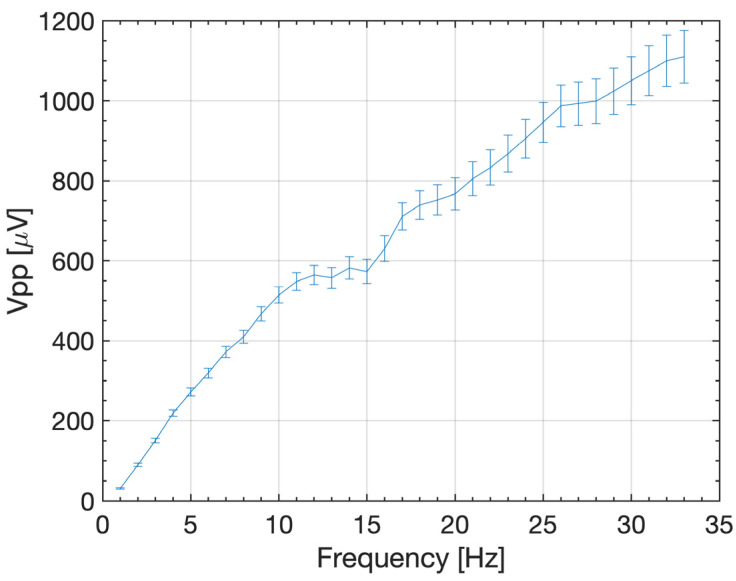
Peak-to-peak voltage induced in the pick-up coil against the vibrational shaker driving frequency for the ferrofluid plug with experimental error.

Figure 4 shows the *V_PP_* voltage induced in the pick-up coil against the vibrational shaker driving frequency for the ferrofluid plug. The *V_PP_* voltage shows a steep increase until the 11 Hz point, then a flatter gradient is observed around 12–16 Hz. Beyond 16 Hz, the graph returns to a slower rate of increase until 33 Hz with a maximum *V_PP_* of 1.1 mV. This can be attributed to the dynamics of the liquid energy harvesting devices. The change in frequency affects the velocity that the fluid oscillates and relates the velocity to the peak-to-peak voltage (Equation (4)). Characterizing the relationship between the induced voltage velocity of the ferrofluid provides insight into how the system operates with different sources of vibrations and frequencies. Hence, a Basler (Basler AG, Ahrensburg, Germany) acA1440-220um camera with a full frame sampling frequency of 160 Hz was used to study the fluid motion.

**Figure 5:**
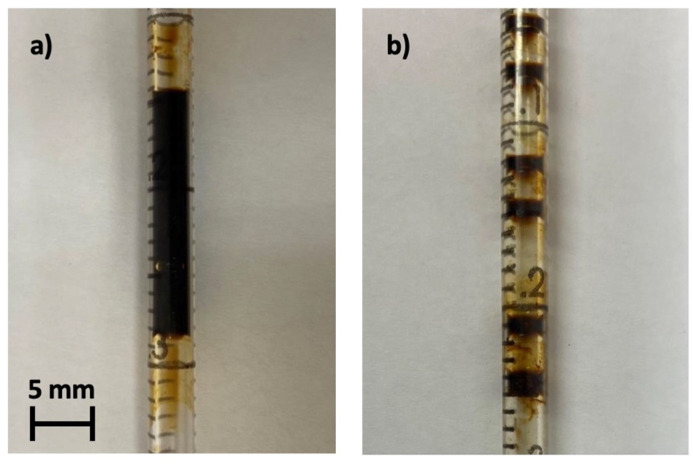
Example photo of the ferrofluid plug inside pipette (**a**) and after the ferrofluid plug has been pulled apart (**b**).

It has been observed that for frequencies >33 Hz, the ferrofluid plug splits into smaller plugs leading to an immediate decline in voltage. Figure 5 shows an example photo of the stationary ferrofluid plug inside pipette (a) and after the ferrofluid plug has been pulled apart into smaller plugs (b). The ferrofluid plug was continuous (Figure 5a) below frequencies of approximately <33 Hz and split into six smaller ferrofluid plugs after being exposed to a frequency >33 Hz. The authors observed the effect of splitting plugs into multiple smaller plugs and spreading along the tube once the drive frequency exceeded approximately 33 Hz in capillaries without an external magnet and filled with pure water. Hence, it can be concluded that the separation is purely a mechanical effect determining the high-frequency limit of the proposed water-based ferrofluid energy harvesting device.

**Figure 6:**
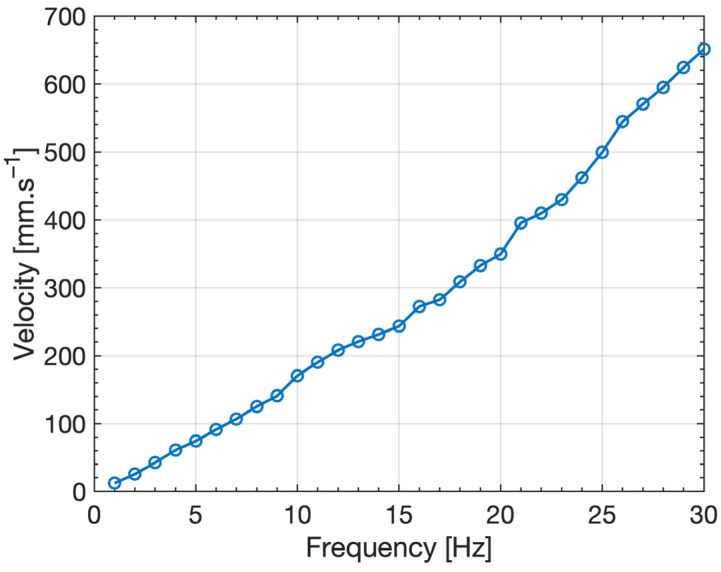
Mean velocity of the ferrofluid plug induced by the shaker against the driving frequency of the vibrational shaker.

Figure 6 shows the mean velocity of the ferrofluid plug induced by the shaker against the driving frequency of the vibrational shaker. For increasing oscillation frequencies, the velocity of the ferrofluid plug, which therefore increases the changes in magnetic flux, linearly increases. Consequently, as the change in magnetic flux increases, so does the induced voltage (Figure 4). At frequencies >33 Hz, the force exerted on the ferrofluid during this change of direction is greater than the surface tension within the liquid, resulting in the plug being pulled apart and splitting into multiple smaller plugs (Figure 5b). This can be attributed to the incising velocity of the ferrofluid plug with increasing frequencies and subsequently increasing changes in momentum due to the oscillating nature of the device. This system is, therefore, unsuitable for use in scenarios where the vibration frequencies exceed 33 Hz but can reliably operate at a lower frequency below the threshold. On the other hand, it is assumed that a ferrofluid plug with a higher surface tension could sustain higher frequencies.

**Figure 7:**
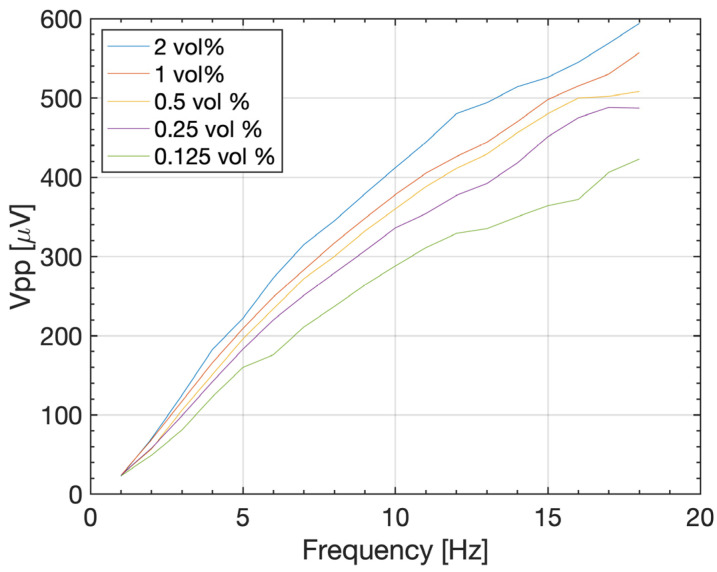
Peak-to-peak voltage for various ferrofluid concentrations.

Figure 7 shows peak-to-peak voltage for ferrofluid concentrations of 2 vol%, 1 vol%, 0.5 vol%, and 0.25 vol%. The generated voltage depends on the alternating magnetic flux linkage in the inductive coil (Equation (3)), which is caused by the permeability of the ferrofluid. Diluting the ferrofluid reduces the iron oxide nanoparticles concentration leading to fewer magnetic particles and, therefore, a lower permeability of the fluid. Subsequently, the reduced permeability of the ferrofluid leads to a reduction in *V_PP_* induced in the pick-up coil. Diluting the ferrofluid from 2 vol% to 1 vol% reduces *V_PP_* from 420 µV to 380 µV. On the other hand, it is assumed that increasing the concertation of the ferrofluid would increase the voltage output of the energy harvesting device. This provides an interesting optimization approach for upscaling this device by utilizing ferrofluids with higher concentrations and higher surface tension γ (Equation (1)) for larger changes in magnetic flux. Future work will focus on stable concentrations of high-density ferrofluids in order to harvest more electrical energy from external vibrations. Future work will also focus on the fluid dynamic analysis (e.g., friction, drag, resonance) to better understand why the plug splits into smaller plugs and on random vibrations sources, which has not been explored for reasons of simplicity. On the other hand, the unique advantage of the here proposed energy harvesting design is the not-reliance on resonance operation conditions. Subsequently, it does not need to conform to certain geometries and masses as long as capillary action is possible. In addition, the vibration source can have any confined shape as long as it can be transferred into a pulsating change in volume. When scaling the proposed energy harvesting device, multiple magnet-pick-up coil assemblies can be placed on a single capillary tube significantly improving the energy harvesting output.

Table 1 compares previously reported energy harvesting devices based on their maximum voltage and maximum frequency. Most devices operate below 10 Hz [10,13,15,18] with voltages between 9–31 mV. On the other hand, generators that operate at a frequency of 30 Hz tend to have a voltage of around 1 mV showing an inverse relationship between voltage and frequency for this type of liquid energy harvesting device. When utilizing a sloshing column of ferrofluid, the maximum voltages generated by our device are smaller, 1.04 mV, compared to previously used cylindrical containers generating 31 mV [18]. The capillary design in this work only uses 1 mL of ferrofluid compared to over 400 mL in the cylindrical container design providing substantial advantages in size and energy density. Compared to the previously prosed pulsating ferrofluid pipe design with a 1000-turn solenoid [24], the here proposed 200-turn solenoid is scalable (Equation (4)). It also utilizes harmonic vibrations instead of a continuous fluid flow with random frequency voltage peaks [23]. This gives the proposed design the advantage of being more easily utilized in real-life scenarios. The capillary design also has the advantage of not depending on resonance effects for operation, meaning it does not have a single operating frequency for a fixed geometry.

## 4. Conclusions

The proposed proof-of-concept liquid vibration energy harvesting device utilizes capillary action with ferrofluids and demonstrates a feasible way of converting pressure differences into electrical signals. The device reliably converts vibrational mechanical energy to electrical energy by generating voltages with magnitudes of up to 1.1 mV. It was found that increasing the frequency of the vibration source led to an increase in the voltage induced in the pick-up coil; however, beyond a frequency >33 Hz, the ferrofluid plug split apart into smaller plugs, and the device broke down, no longer converting energy. Additionally, it was discovered that at higher frequencies, the shape of the voltage signal becomes more distorted compared to the harmonic cycles at low frequencies. It is concluded that the ferrofluid energy harvesting device continuously converts mechanical pressure variations between 1–33 Hz into an AC voltage of identical frequency available for discharge. It can also be concluded that any change in ferrofluid particle concentration directly affects the voltages generated. Compared to existing ferrofluid energy harvesting devices using containers, the proposed device is designed minimally, only requiring a ferrofluid-filled capillary with a magnet-pick-up coil assembly leading to greater energy harvesting capabilities compared to previously reported devices. Future work will focus on the dynamics of the energy harvesting device and explore an array of ferrofluid plugs to scale and maximize power.

## Figures and Tables

**Table 1:** Peak voltage and frequency for ferrofluid energy harvesting devices presented in the literature.

Peak Voltage [mV]	Frequency at Peak Voltage [Hz]	Reference [-]
31.0	1.9	[18]
18.0	9.0	[10]
9.0	5.0	[13]
2.1	6.0	[15]
0.9	30.0	[24]
1.1	30.0	This work
